# Relationship of PSC to embryos: Extending and refining capture of PSC lines from mammalian embryos

**DOI:** 10.1002/bies.202400077

**Published:** 2024-10-14

**Authors:** Qi‐Long Ying, Jennifer Nichols

**Affiliations:** ^1^ Eli and Edythe Broad CIRM Center for Regenerative Medicine and Stem Cell Research at USC, Keck School of Medicine University of Southern California Los Angeles California USA; ^2^ MRC Human Genetics Unit, Institute for Genetics and Cancer University of Edinburgh Edinburgh UK

**Keywords:** blastocyst, chimeras, diapause, epiblast, ESC derivation, germline transmission, naive pluripotent human ESCs

## Abstract

Pluripotent stem cell lines derived from preimplantation mouse embryos have opened opportunities for the study of early mammalian development and generation of genetically uncompromised material for differentiation into specific cell types. Murine embryonic stem cells are highly versatile and can be engineered and introduced into host embryos, transferred to recipient females, and gestated to investigate gene function at multiple levels as well as developmental mechanisms, including lineage segregation and cell competition. In this review, we summarize the biomedical motivation driving the incremental modification to culture regimes and analyses that have advanced stem cell research to its current state. Ongoing investigation into divergent mechanisms of early developmental processes adopted by other species, such as agriculturally beneficial mammals and birds, will continue to enrich knowledge and inform strategies for future in vitro models.

## Graphical abstract

ESCs were first derived from mouse diapause blastocysts or isolated ICMs plated onto fibroblast feeders in a medium containing FBS. Feeders were then replaced with LIF, and later, BMP4 was substituted for serum. GSK3 and ERK inhibition finally enabled efficient derivation of ESCs from embryos of all strains of mice and rats.

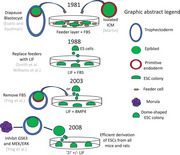

## ADVENT OF NAÏVE PLURIPOTENT STEM CELL LINES

The derivation, characterization, and application for biomedical purposes of pluripotent stem cell lines from mammalian embryos over the past four decades have revolutionized approaches to understanding developmental processes and dysfunction. The first embryonic stem cell (ESC) lines were derived directly from preimplantation mouse embryos, utilizing conditions previously optimized to facilitate self‐renewal of undifferentiated embryonal carcinoma cells in a medium supplemented with fetal bovine serum (FBS) in dishes coated with a layer of mitotically inactivated fibroblast “feeder” cells.^[^ ^1^, ^2^ ^]^ The resulting discrete, dome‐shaped colonies can be propagated clonally when disaggregated, diluted, and replated, retaining the identity and developmental potential of the preimplantation epiblast. This characteristic defines this state of pluripotency as “naïve”.^[^ ^3^ ^]^ Initially, ESCs were derived from mice of the 129 strain, which are coincidentally prone to germline tumors.^[^ ^4^ ^]^ To increase the efficiency and applicability of ESC derivation, efforts were focused to reduce poorly controlled variables, such as the feeder layer. Replacing established fibroblast cell lines with early passage mitotically inactivated murine embryonic fibroblasts (MEFs) in FBS‐containing medium facilitated capture, albeit with low efficiency, of ESCs from some non‐129 strains of mice, such as C57BL/6,^[^ ^5^ ^]^ the background of choice for immunologists and researchers into hematopoiesis. Medium conditioned by buffalo rat liver (BRL) feeder cells enabled self‐renewal of ESCs.^[^ ^6^ ^]^ The “differentiation inhibiting activity” was identified as leukemia inhibitory factor (LIF), small volumes of which could replace feeder cells for propagation of ESCs from the 129 strain^[^ ^7^, ^8^ ^]^ and derivation from 129 embryos poised in the state of peri‐implantation by means of diapause,^[^ ^9^ ^]^ pioneered for one of the original ESC derivation achievements.^[^ ^2^ ^]^ Diapause is a simple way to maintain the epiblast in an optimal state, exposed to minimal growth signals and metabolic activity, from which it may resume normal development in utero, or respond to signals in culture when placed in a permissive environment. This prolonged state of pluripotency allows for more efficient isolation and establishment of ESC lines, as the cells remain capable of self‐renewal.^[^ ^3^, ^10^ ^]^ 

The exploitation of diapause to sustain epiblast in its naïve state of pluripotency, coupled with skillful micro‐dissection to remove extra‐embryonic tissues, thereby eliminating specific differentiation‐inducing signals, promoted highly efficient derivation of ESCs from 129 mice, and variable success from other strains, including the hitherto recalcitrant CBA, but not the non‐obese diabetic (NOD) strain.^[^ ^11^ ^]^ Removal of diapause embryos from the uterus rapidly relieves the imposed inhibition to developmental progression. Differentiation‐inducing signals in the context of early embryonic development are primarily mediated by extra‐embryonic tissues, such as the trophectoderm and extra‐embryonic endoderm, which secrete signaling molecules including FGFs, TGF‐β, and Wnt family members. These drive the inner cell mass (ICM) cells toward specific lineages and away from the pluripotent state.^[^ ^12^, ^13^ ^]^ Analysis of lineage morphology during culture of diapause embryos revealed that strain 129 epiblasts enlarged significantly compared with the less permissive C57BL/6 and CBA, which consequently became dominated by the overlying primitive endoderm/hypoblast.^[^ ^14^ ^]^ The increased epiblast size of 129 diapause embryos obviously provides more cells from which to attempt derivation of ESCs, but its efficiency may also reflect intrinsic differences in cell proliferation or survival, independent of the influence of extra‐embryonic tissues. The potential mechanisms responsible for the privileged state of 129 epiblasts have been at least partially attributed to its more efficient response to STAT3 signaling compared with less permissive strains.^[^ ^15^, ^16^ ^]^ Interestingly, the absence of STAT3, the downstream‐effector of LIF signaling,^[^ ^17^ ^]^ in diapause embryos causes catastrophic loss of the epiblast within a few days of ovariectomy.^[^ ^18^ ^]^ This highlights the crucial role of STAT3 in maintaining epiblast viability, pluripotency, and self‐renewal in vivo during a period of developmental arrest.

## REPLACEMENT OF FBS WITH DEFINED FACTORS

The requirement for FBS as a supplement for culture and derivation of ESCs introduces variability, requiring researchers meticulously to test batches before embarking upon experiments. Its removal would maximize consistency and enable dissection of the mechanisms required to maintain self‐renewing naïve pluripotent stem cells, potentially broadening the repertoire of strains and species from which ESCs could be derived. Culturing ESCs in a defined medium without feeders, FBS, or LIF results in differentiation into neurons.^[^ ^19^ ^]^ Provision of LIF alone failed to block this activity. However, in combination with BMP4, which induces inhibitor of differentiation (ID) proteins, differentiation was repressed and ESC self‐renewal supported. Disappointingly, however, derivation was possible only from the 129 strain.^[^ ^20^ ^]^ Inhibition of glycogen synthase kinase 3 (GSK3) by 6‐bromo‐indirubin 3 oxime promoted limited ESC proliferation.^[^ ^21^ ^]^ A more specific GSK3 inhibitor, Chir99021, was combined with inhibition of FGF/MEK/ERK signaling, previously shown to reduce differentiation,^[^ ^22^ ^]^ resulting in a defined medium composition, known as “2i”, in which ESCs could be propagated efficiently with minimal differentiation, whilst retaining germline competence. The addition of LIF enhanced clonal propagation of ESCs in 2i, but was not essential for their derivation, as demonstrated using embryos lacking STAT3.^[^ ^23^ ^]^ The use of 2i + LIF produced ESCs from NOD embryos^[^ ^24^ ^]^ that could undergo two rounds of gene targeting and still transmit through the germline when injected into host blastocysts.^[^ ^25^ ^]^ Provided ESC cultures are passaged in a timely manner and not allowed to become overgrown,^[^ ^26^, ^27^ ^]^ 2i + LIF provides a universal regime for generating, targeting, and transmitting genetic modifications through the mouse germline, as summarized by Mulas et al.^[^ ^28^ ^]^

## STRATEGIES TO CAPTURE THE ELUSIVE NAÏVE PLURIPOTENT STEM CELLS FROM OTHER RODENTS

Following implantation in the uterus, the embryos of both mice and rats organize into “egg cylinders”, which then undergo gastrulation. Despite the apparent similarity in embryonic morphology, attempts to derive naïve pluripotent rat ESCs met with failure until the advent of 2i + LIF.^[^ ^29^, ^30^ ^]^ Although derivation efficiency was high, rat ESCs were unstable, tending to express markers of extra‐embryonic lineages unless the concentration of GSK3 inhibitor was reduced to 1/3 of that used for mouse ESCs.^[^ ^31^ ^]^ Gene targeting of rat ESCs and transmission through the germline was subsequently achieved using 2i medium with reduced GSK3 inhibitor and LIF throughout the culture,^[^ ^32^ ^]^ opening the door to opportunities for the generation of novel rat models for gene function and disease.

Maximal derivation efficiency of mouse ESCs was accomplished by culturing embryos from the morula stage, embryonic day (*E*) 2.5, in KSOM (potassium‐based Simplex Optimized Medium; the original formula engineered to support development to term of mouse embryos cultured from zygote to blastocyst stage before transfer to pseudo‐pregnant recipient females),^[^ ^33^ ^]^ supplemented with inhibitors for GSK3 and MEK/ERK signaling. This resulted in the redirection of the entire ICM, which would normally segregate into epiblast and hypoblast, into a substantial, compact ball of epiblast, readily expandable into ESC lines when disaggregated.^[^ ^34^ ^]^ To attempt to determine the arrival and departure of naïve pluripotency potential in vivo, ICM and epiblast cells were isolated from mouse embryos at various stages and plated individually directly into 2i + LIF culture.^[^ ^35^ ^]^ Cells isolated from morulae could produce naïve pluripotent stem cell lines only if cultured on a permissive substrate, such as laminin or fibronectin, or a mixture of both, as would be encountered within the developing blastocyst. The frequency of clonal naïve pluripotent stem cell line derivation increased from early blastocyst, peaked just before implantation, and was lost, once the postimplantation epiblast epithelialized and acquired a pro‐amniotic cavity.^[^ ^35^ ^]^ During normal development, soon after implantation, the hypoblast differentiates into visceral endoderm that plays an essential role in imposing anterior–posterior patterning on the epiblast.^[^ ^36^, ^37^, ^38^ ^]^ Interestingly, pluripotent stem cell lines capable of differentiating into most adult tissues can be derived from postimplantation murine epiblasts, following their separation from the overlying visceral endoderm, using defined medium supplemented with FGF2 and activin on a layer of fibronectin.^[^ ^39^, ^40^ ^]^ These epiblast stem cell (EpiSC) lines proliferate as flat, epithelial colonies, tend to exhibit low cloning efficiency and diverge from ESCs in several respects, as discussed in Smith, this issue^[^ ^41^ ^]^ and are referred to as “primed” pluripotent stem cells.^[^ ^3^ ^]^ Although they can be derived from pre‐ to late‐gastrulating epiblasts (E5.5 to E7.5), their properties become most closely aligned with the anterior primitive streak tissue of the E7.5 epiblast after serial passaging.^[^ ^42^ ^]^ However, further studies have shown that EpiSCs express high levels of WNT signals,^[^ ^43^ ^]^ which induce differentiation into primitive streak cells. This differentiated subset drives the observed alignment with the primitive streak, whereas undifferentiated EpiSCs, cultured in the presence of WNT inhibitors, more accurately correspond to the pre‐gastrula epiblast.^[^ ^44^, ^45^, ^46^ ^]^ Endeavors to derive EpiSCs were primarily inspired by the properties of human ESCs propagated from surplus blastocysts donated from assisted conception programmes, which require similar culture conditions and grow as flat, epithelial colonies.^[^ ^47^ ^]^ Capturing naïve pluripotent stem cell lines from human embryos turned out not to be straightforward.^[^ ^48^ ^]^ As an essential interim measure, reprogramming strategies were painstakingly applied to primed hESCs, resulting in the long‐awaited self‐renewing naïve pluripotent cultures.^[^ ^49^, ^50^ ^]^

## DERIVATION OF NAÏVE PLURIPOTENT STEM CELL LINES FROM PREIMPLANTATION HUMAN EMBRYOS

Culturing human morulae in 2i or combinations of MEK/ERK and FGF receptor inhibitors, exactly as used for mouse ESC derivation, did not block hypoblast formation.^[^ ^48^, ^51^ ^]^ An alternative means to protect early epiblast cells from instructions to differentiate was to separate ICM cells before plating. The medium optimized for reprogramming primed human stem cells^[^ ^49^ ^]^ enabled the derivation of cell lines, termed “HNES” (Human Naïve Epiblast Stem) cells that proliferated as dome‐shaped colonies that could be passaged clonally and closely resembled human blastocyst cells in gene expression profile.^[^ ^52^ ^]^ An alternative strategy exploiting the medium composition developed for efficient reprogramming of primed hESCs to the naive state^[^ ^50^ ^]^ was also applied to intact donated human blastocysts, resulting in the derivation of novel human stem cell lines exhibiting properties of naïve pluripotency.^[^ ^53^ ^]^

## INTEGRATION OF MOUSE ESCs INTO HOST EMBRYOS

Mouse ESCs, genetically modified using ever‐evolving engineering technology, can be used to generate biomedically relevant mouse models, following incorporation into host wildtype preimplantation embryos, transfer to recipient females, and transmission through the germline, as first demonstrated four decades ago.^[^ ^54^ ^]^ Modifications to ESC culture regimes and embryo stage have resulted in the establishment of cell lines variously referred to as “expanded potential” or “extended pluripotent” stem cells, reported to contribute to extraembryonic lineages of developing embryos.^[^ ^55^, ^56^ ^]^ Additional studies have recounted the contribution of derivatives of ESCs exhibiting 2‐cell stage‐like identity, attributed to retroviral influences,^[^ ^57^ ^]^ or ESCs selected for expression of a highly sensitive Hex reporter^[^ ^58^ ^]^ to trophoblast and/or hypoblast when injected into host embryos. The properties and utility of such cell lines have been discussed comprehensively in a recent, excellent review.^[^ ^59^ ^]^

## USING CHIMERAS TO STUDY CELL COMPETITION

Soon after implantation in the uterus, the mouse epiblast undergoes rapid cell division in preparation for gastrulation. It was subsequently reported, via single‐cell RNA sequencing of embryos treated with a cell death inhibitor, that around 35% of epiblast cells undergo apoptosis at this time, which is proposed to serve as a means to eliminate unsuitable cells from participating in the formation of embryonic tissues, including those that will give rise to the germ cells.^[^ ^60^ ^]^ Evidence for the existence of mechanisms that implement destruction and elimination of cells somehow perceived by the early postimplantation epiblast to be “less fit” than their neighbors was obtained by the use of chimeras created by injection of ESCs carrying abnormalities that, in the context of the intact embryo, would be viable. Examples of defects carried by ESCs that were found to be selectively instructed to apoptose include those with mutations in BMP signaling, defects in autophagy, and possession of a tetraploid genome.^[^ ^61^ ^]^ Several years of further research revealed roles for mTOR, its upstream activator, p53, and defects in mitochondrial processes as readouts for cell fitness that could begin to explain how death or survival could be determined for each cell within a mixed population.^[^ ^60^, ^62^ ^]^ Elevated levels of the proto‐oncogene cMyc had previously been proposed to confer enhanced cell fitness on epiblast cells during murine gastrulation.^[^ ^63^ ^]^ Interestingly, such a mechanism is not invoked during the blastocyst stage of development to select 1–3 of the 10–15 ESCs generally injected prior to embryo transfer to secure contribution to chimeric offspring.^[^ ^64^ ^]^ ESCs lacking cMyc are able to persist during preimplantation stages within wild‐type host embryos.^[^ ^65^ ^]^ It appears that deviating from the naïve state of pluripotency, as befalls ESCs cultured in the serum‐containing medium compared with 2i + LIF, induces cell death in injected cells as early as the morula stage.^[^ ^65^ ^]^ This is surprising since blastomeres at this stage are considered to be “unspecified”. So far, the mechanisms by which the unsuitable (non‐naïve pluripotent) injected ESCs are identified and executed have not been uncovered.

## SELF‐ORGANIZATION OF ESCs TO MIMIC DEVELOPMENTAL PROCESSES

A significant motivation for creating pluripotent stem cell lines was to model aspects of early mammalian development and produce tissues by directed differentiation, as presented in several chapters in this issue.^[^ ^66^, ^67^, ^68^ ^]^ The derivation of trophoblast stem cells (TSCs)^[^ ^69^ ^]^ and extraembryonic endoderm (XEN) cells^[^ ^70^ ^]^ has been instrumental in advancing various research applications. However, these early attempts produced cell types that corresponded more closely to postimplantation stages, rendering them unsuitable for the generation of 3D structures resembling blastocysts, known as “blastoids”. Recent endeavors have instead focused on deriving pre‐implantation stage trophectoderm and primitive endoderm stem cell lines, which are more appropriate for creating blastoids.^[^ ^71^, ^72^, ^73^ ^]^ In the early attempts to create mouse blastoids, TSCs were combined with ESCs, which approximated blastocysts morphologically, but lacked sufficient hypoblast/primitive endoderm tissue and, although able to implant in the uterus, failed to initiate normal development.^[^ ^74^ ^]^ The mouse system was chosen for this study to develop and validate a protocol that could then be adapted to generate blastoids from human stem cell lines to model the less accessible human early development. Fortuitously, however, human naïve pluripotent stem cell lines were found to exhibit greater developmental flexibility compared with their murine counterparts and can readily differentiate into trophoblast or hypoblast by minimal modifications to the culture.^[^ ^75^ ^]^ Production of human blastoids was therefore relatively straightforward.^[^ ^76^, ^77^ ^]^ As with mouse blastoids, the formation of human hypoblast tended to be somewhat restricted, but by using sequential media modifications it can be titrated to produce more realistic blastocyst models.^[^ ^78^ ^]^ The production and use of blastoids is described in detail in Smith, this issue.^[^ ^41^ ^]^

In addition to their worldwide use as a means to transmit genetic modifications through the mouse germline via injection into preimplantation mouse embryos and transfer to a pseudo‐pregnant host uterus,^[^ ^54^, ^79^ ^]^ pluripotent stem cells have been adopted for modeling developmental processes and postimplantation embryos in vitro. Early hypotheses speculating how the pro‐amniotic cavity may form were tested using embryoid bodies, which mimic an epiblast surrounded by a layer of endoderm‐like epithelium.^[^ ^80^ ^]^ These pioneering studies were subsequently questioned and tested using more advanced technology,^[^ ^81^, ^82^ ^]^ which did not support the original conclusion that cell death in the center of the “epiblast” region was induced by signals from the overlying endoderm‐like epithelium. Self‐organizing ESC models have also been developed in which signaling gradients, polarization, and formation of a primitive‐streak‐like region have been observed, which laid the groundwork for more complex models.^[^ ^83^ ^]^ The evolving development of ESC‐derived structures mimicking posterior postimplantation development, known as “gastruloids”,^[^ ^84^, ^85^, ^86^ ^]^ are expertly discussed by Turner and Martinez Arias in this issue.^[^ ^68^ ^]^

## FUTURE PERSPECTIVES

In the current landscape, chimera‐forming and germline‐competent naïve PSCs have been successfully derived from mice and rats, marking significant advancements. However, this achievement has not extended to many other species. The establishment of germline‐competent PSCs from non‐rodent species holds tremendous scientific and therapeutic promise. We anticipate that the underlying principles governing the capture and maintenance of naïve pluripotency across species are likely shared, albeit with nuanced differences attributable to variations in early embryonic development. The potential avenues for developing conditions to capture and sustain naïve pluripotency are manifold. The well‐established mouse and rat ESCs provide a conducive environment for easily discerning and dissecting signaling pathways. As elaborated above, the 2i condition, initially developed for mouse ESCs,^[^ ^23^ ^]^ was successfully formulated to derive the first rat ESCs.^[^ ^29^, ^30^ ^]^ Emphasizing this point, utilizing rat ICMs initially instead of mice for such research would likely have posed a formidable challenge. Further exploration of fate determination in preimplantation embryos, particularly understanding the intricacies involved in lineage specification and inhibition, may provide insights to pave the way for developing conditions conducive to maintaining naïve ESCs in a broader range of species. The Supporting Information table summarizes the main scientific milestones presented in this study.

## AUTHOR CONTRIBUTIONS

Both authors participated in some advances of protocol development, production, and analysis of pluripotent stem cell lines. This essay was written as an equal collaboration.

## CONFLICT OF INTEREST STATEMENT

The authors have no conflict of interest to declare.

## Supporting information



Supporting Information

## Data Availability

Data sharing is not applicable to this article as no new data were created or analyzed in this study.
