# Supplementary material for: Relationship of PSC to embryos: Extending and refining capture of PSC lines from mammalian embryos
Source: Bioessays. 2024 Oct 14;46(12):2400077. doi: 10.1002/bies.202400077 (PMC11589693; doi:10.1002/bies.202400077)
Supplement: Supplementary file 1 — Supporting Information [file BIES-46-2400077-s001.docx]

| **Experimental advance** | **Unanticipated feature/obstacle** | **Reference** |
| --- | --- | --- |
| Isolation of pluripotent stem cell lines from testicular teratomas | Tendency for cell lines to carry karyotypic abnormalities | [1,2] |
| Derivation of embryonic stem cells (ESC) directly from preimplantation mouse embryos | Original culture required strain 129 mice, already in place in host labs owing to their tendency for germline tumours | [3]  [4] |
| Production of ESCs from C57BL/6 mouse embryos | Low efficiency | [5] |
| Replacement of feeders with factor isolated from buffalo rat liver (BRL) cells | First labelled as ‘Differentiation Inhibitory Activity’, the factor was subsequently identified as ‘Leukaemia Inhibitory Factor’ (LIF) | [6-8] |
| Massive improvement in ESC derivation efficiency, including previously recalcitrant strains using epiblasts dissected from diapause embryos | Epiblast dissection from peri-implantation or diapause embryos requires considerable skill in micromanipulation | [9] |
| ESCs derived from human embryos exhibited epithelial properties of postimplantation epiblasts | Unlike naïve pluripotent mouse ESCs, FGF signalling was required for survival of the original human embryonic stem cell lines | [10] |
| Induced pluripotent stem cells generated by transcription factor-mediated reprogramming of somatic mouse cells | Transfection of *Oct4, Sox2, KLF4* and *Myc,* selected by reductive transfection of ECAT factors into fibroblasts, enabled conversion to the naïve state and self-renewal of pluripotent cells | [11] |
| Derivation of epiblast stem cell (EpiSC) lines from postimplantation mouse epiblasts | To assist researchers restricted from accessing human pluripotent stem cells, representative lines were obtained from mouse using the human ESC derivation medium | [12,13] |
| Importance of STAT3 signalling demonstrated for maintenance of epiblast, particularly in 129 embryos/ESCs | Epiblasts of diapause 129 embryos proliferate more robustly in culture than other strains; they are more responsive to LIF/STAT3 signalling, which is required to maintain the entire ICM during diapause | [14]  [3,15]  [16] |
| Development of strategy to replace feeders and serum for efficient production of ESCs from all mouse strains | Blocking GSK3 and MEK/ERK signalling in defined medium is sufficient to enable derivation of ESCs; addition of LIF enhances clonability | [17-19] |
| Derivation of first *bona fide* ESCs from rat embryos | Despite similar embryology to mouse, rat ESCs could not be derived until the advent of 2i+LIF | [20]  [21] |
| Inhibition of MEK/ERK signalling at the mouse morula stage enhances ESC derivation efficiency | FGF signalling was found to be necessary and sufficient for induction of hypoblast in mouse embryos | [18]  [22] |
| Naïve pluripotent stem cell lines derived from human preimplantation embryos | Inhibition of multiple factors enabled derivation of naïve pluripotent stem cell lines from human epiblasts | [23]  [24] |
| Injection of mouse ESCs to host blastocysts for investigation of potency, making mouse models for lineage analysis, gene function and biomedicine | Use of mouse chimeras identified mechanisms of cell competition as a checkpoint to eliminate cells unfit for gastrulation, accounting for around 35% of epiblast cells | [25]  [26] |
| Using ESCs to form self-organising structures resembling blastocysts | Human naïve pluripotent stem cells easily form trophectoderm and hypoblast, simplifying blastoid formation compared with those from mouse ESCs | [27]  [28]  [29]  [30] |

1 **Rosenthal MD, Wishnow RM, Sato GH.** 1970. In vitro growth and differetiation of clonal populations of multipotential mouse clls derived from a transplantable testicular teratocarcinoma. *J Natl Cancer Inst* **44**: 1001-14.

2 **Kahan BW, Ephrussi B.** 1970. Developmental potentialities of clonal in vitro cultures of mouse testicular teratoma. *J Natl Cancer Inst* **44**: 1015-36.

3 **Evans MJ, Kaufman M.** 1981. Establishment in culture of pluripotential cells from mouse embryos. *Nature* **292**: 154-6.

4 **Martin GR.** 1981. Isolation of a pluripotent cell line from early mouse embryos cultured in medium conditioned by teratocarcinoma stem cells. *ProcNatlAcadSciUSA* **78**: 7634-8.

5 **Suemori H, Nakatsuji N.** 1987. Establishment of the Embryo-derived Stem (ES) Cell Lines from Mouse Blastocysts: Effects of the Feeder Cell Layer. *Dev Growth Differ* **29**: 133-9.

6 **Smith AG, Hooper ML.** 1987. Buffalo rat liver cells produce a diffusible activity which inhibits the differentiation of murine embryonal carcinoma and embryonic stem cells. *Dev Biol* **121**: 1-9.

7 **Smith AG, Heath JK, Donaldson DD, Wong GG, et al.** 1988. Inhibition of pluripotential embryonic stem cell differentiation by purified polypeptides. *Nature* **336**: 688-90.

8 **Williams RL, Hilton DJ, Pease S, Willson TA, et al.** 1988. Myeloid leukaemia inhibitory factor maintains the developmental potential of embryonic stem cells. *Nature* **336**: 684-7.

9 **Brook FA, Gardner RL.** 1997. The origin and efficient derivation of embryonic stem cells in the mouse. *Proc Natl Acad Sci U S A* **94**: 5709-12.

10 **Thomson JA, Itskovitz-Eldor J, Shapiro SS, Waknitz MA, et al.** 1998. Embryonic stem cell lines derived from human blastocysts. *Science* **282**: 1145-7.

11 **Takahashi K, Yamanaka S.** 2006. Induction of pluripotent stem cells from mouse embryonic and adult fibroblast cultures by defined factors. *Cell* **126**: 663-76.

12 **Brons IG, Smithers LE, Trotter MW, Rugg-Gunn P, et al.** 2007. Derivation of pluripotent epiblast stem cells from mammalian embryos. *Nature* **448**: 191-5.

13 **Tesar PJ, Chenoweth JG, Brook FA, Davies TJ, et al.** 2007. New cell lines from mouse epiblast share defining features with human embryonic stem cells. *Nature* **448**: 196-9.

14 **Batlle-Morera L, Smith A, Nichols J.** 2008. Parameters influencing derivation of embryonic stem cells from murine embryos. *Genesis*.

15 **Ohtsuka S, Niwa H.** 2015. The differential activation of intracellular signaling pathways confers the permissiveness of embryonic stem cell derivation from different mouse strains. *Development* **142**: 431-7.

16 **Kraunsoe S, Azami T, Pei Y, Martello G, et al.** 2023. Requirement for STAT3 and its target, TFCP2L1, in self-renewal of naive pluripotent stem cells in vivo and in vitro. *Biol Open* **12**.

17 **Ying QL, Wray J, Nichols J, Batlle-Morera L, et al.** 2008. The ground state of embryonic stem cell self-renewal. *Nature* **453**: 519-23.

18 **Nichols J, Silva J, Roode M, Smith A.** 2009. Suppression of Erk signalling promotes ground state pluripotency in the mouse embryo. *Development* **136**: 3215-22.

19 **Nichols J, Jones K, Phillips JM, Newland SA, et al.** 2009. Validated germline-competent embryonic stem cell lines from nonobese diabetic mice. *Nat Med* **15**: 814-8.

20 **Li P, Tong C, Mehrian-Shai R, Jia L, et al.** 2008. Germline competent embryonic stem cells derived from rat blastocysts. *Cell* **135**: 1299-310.

21 **Buehr M, Meek S, Blair K, Yang J, et al.** 2008. Capture of authentic embryonic stem cells from rat blastocysts. *Cell* **135**: 1287-98.

22 **Yamanaka Y, Lanner F, Rossant J.** 2010. FGF signal-dependent segregation of primitive endoderm and epiblast in the mouse blastocyst. *Development* **137**: 715-24.

23 **Guo G, von Meyenn F, Santos F, Chen Y, et al.** 2016. Naive Pluripotent Stem Cells Derived Directly from Isolated Cells of the Human Inner Cell Mass. *Stem Cell Reports* **6**: 437-46.

24 **Pastor WA, Chen D, Liu W, Kim R, et al.** 2016. Naive Human Pluripotent Cells Feature a Methylation Landscape Devoid of Blastocyst or Germline Memory. *Cell Stem Cell* **18**: 323-9.

25 **Sancho M, Di-Gregorio A, George N, Pozzi S, et al.** 2013. Competitive interactions eliminate unfit embryonic stem cells at the onset of differentiation. *Dev Cell* **26**: 19-30.

26 **Lima A, Lubatti G, Burgstaller J, Hu D, et al.** 2021. Cell competition acts as a purifying selection to eliminate cells with mitochondrial defects during early mouse development. *Nat Metab* **3**: 1091-108.

27 **Guo G, Stirparo GG, Strawbridge SE, Spindlow D, et al.** 2021. Human naive epiblast cells possess unrestricted lineage potential. *Cell Stem Cell* **28**: 1040-56 e6.

28 **Yanagida A, Spindlow D, Nichols J, Dattani A, et al.** 2021. Naive stem cell blastocyst model captures human embryo lineage segregation. *Cell Stem Cell* **28**: 1016-22 e4.

29 **Kagawa H, Javali A, Khoei HH, Sommer TM, et al.** 2022. Human blastoids model blastocyst development and implantation. *Nature* **601**: 600-5.

30 **Rivron NC, Frias-Aldeguer J, Vrij EJ, Boisset JC, et al.** 2018. Blastocyst-like structures generated solely from stem cells. *Nature* **557**: 106-11.
